# 

**Published:** 1840-07

**Authors:** 


Vol I.—No. VI.

EDITED BY
CHAPIN A. HARRIS, M. D.
Baltimore.
ELEAZAR PARMLY,
New-York.

PUBLISHING COMMITTEE,
E. PARMLY, | E. BAKER,
S. BROWN.

NEW YORK,

GEORGE ADLARD, 1C8 BROADWAY,

BALTIMORE,

ARMSTRONG & BERRY.

PRICE---THREE DOLLARS PER ANNUM.

J. C. Kelley, Printer.

